# A Good Beginning

**Published:** 1902-03

**Authors:** 


### A Good Beginning.

The Italian Bishop of Fano, says the *Chicago Clinic and Pure Water Journal,* has issued the following circular:

1. In all churches, immediately after feast days the floors must be disinfected by means of wood sawdust soaked in one-tenth per cent. of corrosive sublimate. At all times the floors must be frequently swept after sprinkling with water.

2. Every week day the pews and confessionals must be cleansed with pure water.

3. Every week day, and even oftener, the grills of the confessionals must be thoroughly cleansed.

4. The holy water receptacles must be emptied at least once a week, scoured and sterilized. R.

---

*The St. Paul Medical Journal,* in commenting upon the failure of the *American Gynecological and Obstetrical Journal,* with $30,000 in unpaid subscription, says: "A business-like effort should be made to collect subscription accounts, if possible, in advance, and at any rate during the current year, and subscribers who do not pay should be dropped. We can not understand why some medical men, often the very ones who complain the loudest about patients not paying them for their services, are so slow in paying their own bills. It has been our observation that those physicians who are known in their own communities to pay their bills promptly have the smallest number of unpaid accounts upon their books, and the reverse is also very apt to be the case." R.

